# The Training-Schools Must Lead

**Published:** 1918-10-26

**Authors:** 


### THE MATRONS' AND SISTERS' DEPARTMENT.

### THE TRAINING-SCHOOLS MUST LEAD.

General satisfaction has been caused by the announcement that the College of Nursing has appointed a committee to examine into the question of nurses' salaries and report thereon. The subject is one which is being curiously mishandled. Poor-Law Guardians, managers of voluntary institutions, and other employers of nurses are apparently guided by no deeper consideration in their proffers of increased salary than that of securing the hands necessary to carry on the day's labour. They want workers. They must have them, in short, but they sometimes seem to care but moderately what prospect opens before the workers when once they have been secured.

The natural consequence of this pressure on employers, unintelligently directed, is to flood the profession with young women who care for nothing but the immediate prospect of good pay even while they are in the category of the unskilled. It is very tempting to be offered good money, free board, lodging and uniform, attractive society and facilities for recreation, besides the rise in social value which attends even the embryo nurse. But this is not all that is required to gather in the kind of candidate most wanted. To attract women of education, with good mental and moral qualities, there must be something higher. There must be a definite career. Such women look beyond the pupil stages and think little of a few extra pounds to spend while they are learning their business. They and those who advise them want to be assured they are entering a profession, not a *cul-de-sac*.

The responsible heads of training-schools are well aware of these things. Their experience enables them to tell what inducements react best on the class they seek to attract. Where the influence of the matron is allowed full weight it will be found that the salaries of the fourth-year nurses, of the sisters and heads of departments, are considered of far more importance than those of the unskilled, and that a good choice of candidates continues to present itself.

There are still far too many institutions in which the nurses before and after their training is completed are miserably underpaid. The astonishing thing is that they complain so faintly, if at all. Absorbed in duties which are ill-remunerated, with but slender hopes of future advancement, with far too little time off-duty, and yet constantly compelled in the interests of the sick to surrender some part of their scanty privileges, nurses all over the country suffer. In hospitals, Poor-Law infirmaries, district associations, private nursing institutes, nurses are allowing themselves to be exploited. It is an ugly word, but there is no other which expresses so well the extent to which high-minded women of all ranks from the humblest to the highest are being kept out of the emoluments justly due to them by "drivers."

So far as reform is concerned, it is never sufficiently recognised that it is only under the leadership of the women they trust that nurses will consent to move. Trades union agitation in order to put pressure on employers for better conditions of service is offensive to them. Every one of their utterances proves this. It is amazing what they will put up with rather than resort to this weapon. They are dumb. They are patient under glaring abuses. They are easy to oppress. It is indisputable that their conviction of a higher vocation runs directly counter to their worldly interests. Alas, that greedy or thoughtless individuals have been busy turning this selfless zeal to serve selfish ends! But the nurse goes her way unmoved. She sees something real, eternal, most beautiful in her life, unaffected by its outward conditions, and, while those around are trying to rouse her to rebel, she cannot answer. She holds in her heart a secret she cannot betray.

Must things, then, go on as before? Heaven forfend! We see rather in this confidence of nurses in their leaders and in no one else the ground of great hope for the future. All these years the nurses have waited dumb and unrebellious, acquiescing perforce in much which is hard and barren in their lives. Now the signal has been given. Nurses who hear the call are gladly flocking to the banner, for they trust their training-schools. They support the College because it is in accord with all that they value in their professional career. They are certain that their ideals will not be rudely brushed aside by those who instilled these very ideals into their hearts. They look forward with eager faith to the goal.

